# Supplementary material for: An Automated Microwell Platform for Large-Scale Single Cell RNA-Seq
Source: Sci Rep. 2016 Sep 27;6:33883. doi: 10.1038/srep33883 (PMC5037380; doi:10.1038/srep33883)
Supplement: Supplementary Information [file srep33883-s1.pdf]

Supplementary Information for: An Automated Microwell Platform for Large-Scale Single Cell RNA-Seq

Jinzhou Yuan[1] and Peter A. Sims*[1,2,3]

[1]Department of Systems Biology, Columbia University Medical Center, New York, NY 10032 USA
[2]Sulzberger Columbia Genome Center, Columbia University Medical Center, New York, NY 10032 USA
[3]Department of Biochemistry & Molecular Biophysics, Columbia University Medical Center, New York, NY 10032 USA

* Correspondence to pas2182@cumc.columbia.edu

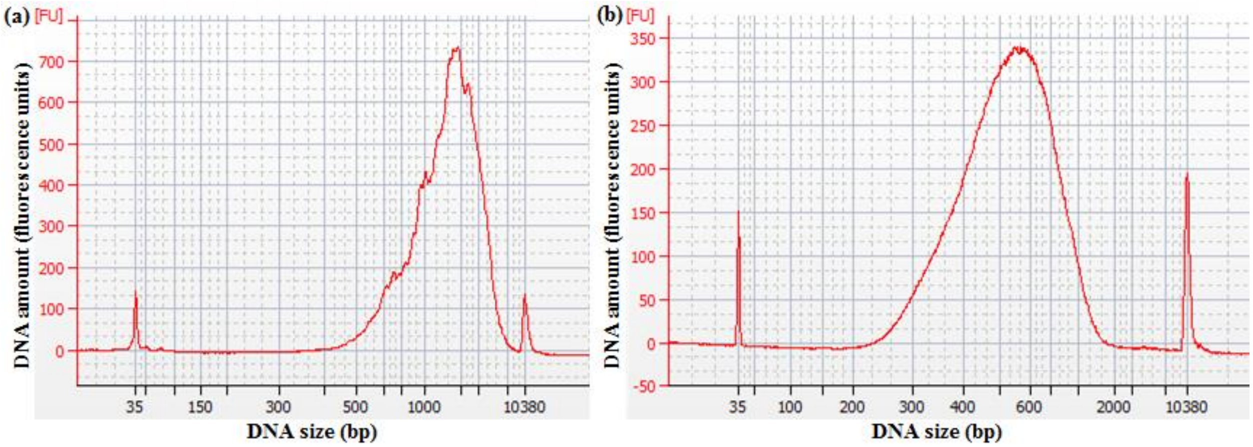

Supplementary Figure S1. Representative Bioanalyzer traces of a) full-length pre-amplified cDNA from the first round of PCR in the library preparation and b) "tagmented" 3'-end sequencing library after PCR enrichment using the Illumina Nextera system.

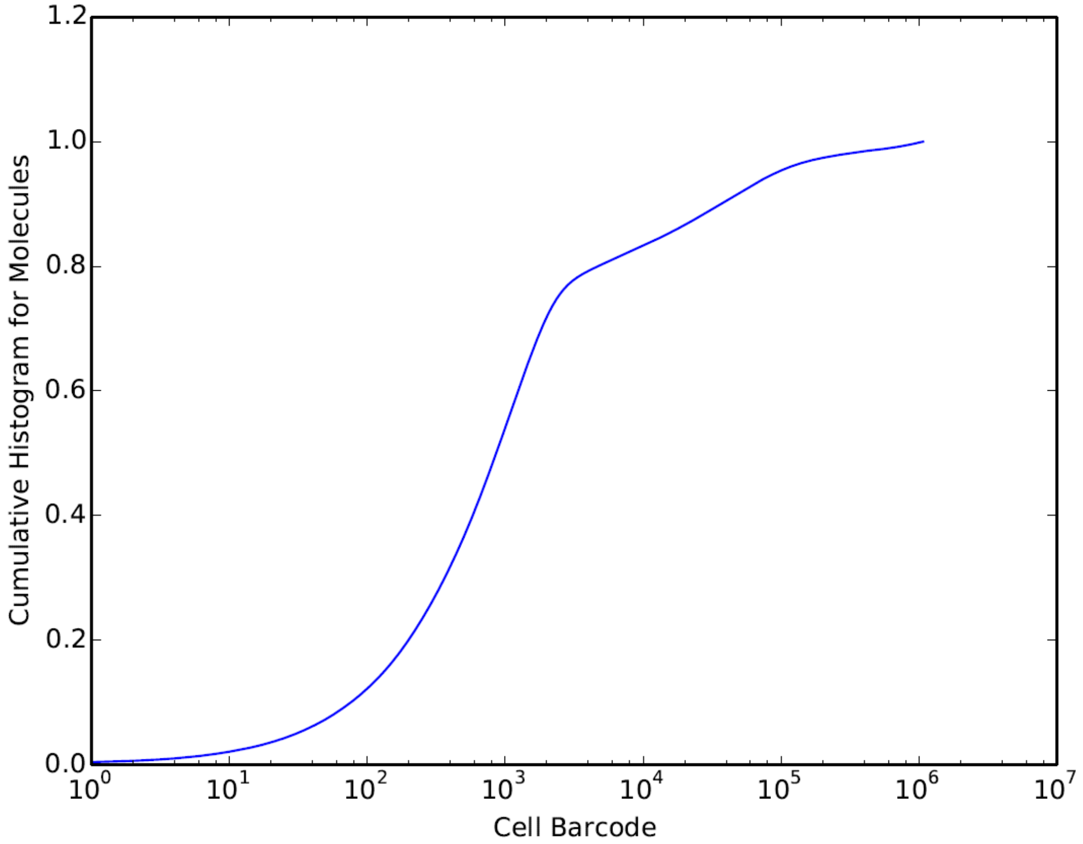

Supplementary Figure S2. Cumulative histogram of the fraction of molecules associated with each detected cell-identifying barcode (see Methods). The histogram shows two major inflection points. The first occurs at ~2,200 cell barcodes which is consistent with the number of cells captured in our device. About 80% of molecules detected are associated with cell-bead pairs in our device. The second occurs at >100,000 cell barcodes. Given that our device holds ~140,000-150,000 beads, we interpret this inflection point as representing cell-identifying barcodes from beads in our device (the majority of which are not associated with a cell). Beads that are unassociated with a cell can still capture a small amount of ambient mRNA due to incomplete washing. We interpret the remaining cell-identifying barcodes past this second inflection point as arising from sequencing or other errors (corresponding to a few percent of the total number of molecules detected).

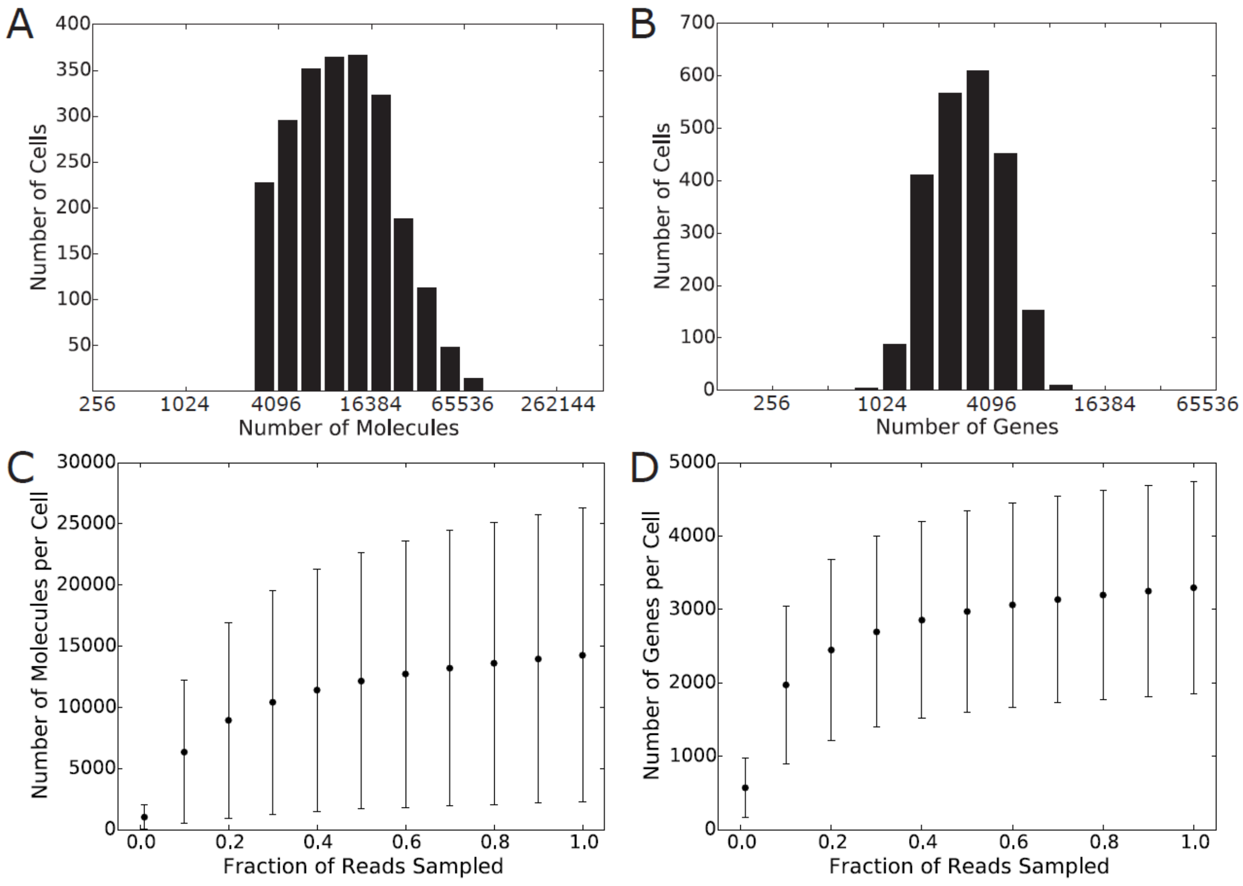

Supplementary Figure S3: **Characterization of Single Cell RNA-Seq Performance on Glioma Neurospheres.** (A) Histogram of the number of uniquely mapped molecules detected per cell based on UMI-filtered reads after error correction. (B) Histogram of the number of genes detected per cell based on uniquely mapped reads. (C) Sub-sampling saturation curve for the number of molecules detected per cell as a function of the number of uniquely mapped reads sampled. (D) Same as (C) for the number of genes detected per cell.

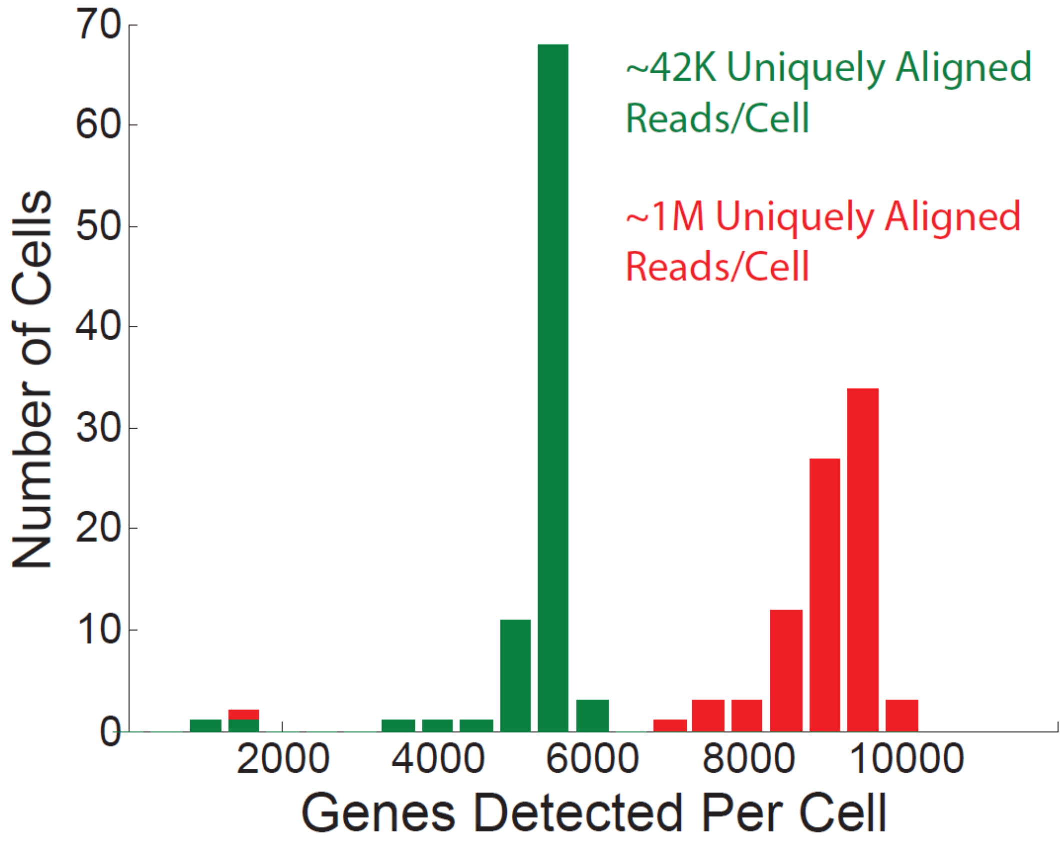

Supplementary Figure S4: **Characterization of Single Cell RNA-Seq Performance of the Fluidigm C1 System on NIH-3T3 Cells (obtained from GSE701151, Macosko et al, *Cell*, 2015).** Histograms of the number of genes detected per 3T3 cell at full coverage (~1 million uniquely aligned reads per cell) and after down-sampling the data to ~42,000 uniquely aligned reads per cell, which is comparable to the sequencing depth used for 3T3 cells in the current study. At full coverage, ~8,800 genes are detected per cell, on average, using the Fluidigm C1 system. At low coverage, ~5,300 genes are detected per cell, on average.

BLUE CLUSTER	RED CLUSTER
CPE	STRF6
XRCC6BP1	ISG15
AGAP2	HIST1H4C
PVT1	NGFR
XIST	S100A4
ANXA1	ID3
VGF	MIA
LOC100131257	BCYRN1
HAPLN1	IFI6
UGDH-AS1	AGRN
VSTM4	S100A1
MGC4836	MT1X
IGFBP3	CCDC80
ZMYND8	ANXA2
BC037952	FCRLA
ZZEF1	FABP7
CBFA2T2	FABP3
MIPOL1	C1orf61
TRIB3	ATP1B2
GRIA2	SOD3
S100A10	KLHDC8A
NKTR	SPARC
KIAA1328	SPARCL1
LOC643406	ARL4C
MALAT1	ATP1A2
HLA-C	S100A6
TLCD2	TSFM
PDE3B	PLAT
UBE2C	YWHAZ
JX088243	NES
GEN1	POLR2K
SBF2	METTL1
KCNQ1OT1	LGALS1
PLK1	NCAN
CCNB1	ZNF706
BC131773	RNF19A
KLF2	MICU3
LOC440354	SLC7A8
DQ582201	FAM49B
GADD45A	VPS37A
CCNL1	CNOT7
MTRNR2L2	ZDHHC2
WSB1	MT1E
Y16709	A2M
TSPAN31	EFR3A

BC018860	AGAP2-AS1
MTRNR2L1	TAGLN2
PILRB	SPAG1
ASPM	
AFG3L1P	
HIST1H1C	
COL20A1	
KPNA2	
TOP2A	
TVAS5	
C1orf63	
PNISR	
SLC25A29	
CSPG4	
MTND5	
JA760602	
PABPC1	
ANKRD12	
CREB5	
HSPA1B	
APOD	
ZAK	
ARMC9	

Supplementary Table S1: List of highly variable genes used to cluster the glioma neurosphere data in **Figure 4**. Genes in the left-most column are, on average, more highly expressed in the blue cluster than the red cluster, and genes in the right-most column are more highly expressed in the red cluster than the blue cluster.

Supplementary Video S1: Multi-round loading of single cells into microwells. Scale bar: 50 μm. Play speed: 10X real time.
